# Long-term p110α PI3K inactivation exerts a beneficial effect on metabolism

**DOI:** 10.1002/emmm.201201953

**Published:** 2013-03-11

**Authors:** Lazaros C Foukas, Benoit Bilanges, Lucia Bettedi, Wayne Pearce, Khaled Ali, Sara Sancho, Dominic J Withers, Bart Vanhaesebroeck

**Affiliations:** 1 Institute of Healthy Ageing and Department of Genetics, Evolution and Environment, University College LondonDarwin Building, Gower Street, London, UK; 2 Centre for Cell Signalling, Barts Cancer Institute, Queen Mary University of LondonCharterhouse Square, London, UK; 3 Promed Laboratoire MédicalMarly, Switzerland; 4 Metabolic Signalling Group, Medical Research Council Clinical Sciences Centre, Imperial CollegeDu Cane Road, London, UK

**Keywords:** aging, cancer, diabetes, insulin signalling, obesity

## Abstract

The insulin/insulin-like growth factor-1 signalling (IIS) pathway regulates cellular and organismal metabolism and controls the rate of aging. Gain-of-function mutations in p110α, the principal mammalian IIS-responsive isoform of PI 3-kinase (PI3K), promote cancer. In contrast, loss-of-function mutations in p110α impair insulin signalling and cause insulin resistance, inducing a pre-diabetic state. It remains unknown if long-term p110α inactivation induces further metabolic deterioration over time, leading to overt unsustainable pathology. Surprisingly, we find that chronic p110α partial inactivation in mice protects from age-related reduction in insulin sensitivity, glucose tolerance and fat accumulation, and extends the lifespan of male mice. This beneficial effect of p110α inactivation derives in part from a suppressed down-regulation of insulin receptor substrate (IRS) protein levels induced by age-related hyperinsulinemia, and correlates with enhanced insulin-induced Akt signalling in aged p110α-deficient mice. This temporal metabolic plasticity upon p110α inactivation indicates that prolonged PI3K inhibition, as intended in human cancer treatment, might not negatively impact on organismal metabolism.

## INTRODUCTION

The insulin/IGF-1 signalling (IIS) pathway plays a major role in growth and metabolism. Phosphoinositide 3-kinases (PI3Ks) are key effector molecules in signal transmission downstream of the insulin/IGF-1 receptor (Vanhaesebroeck et al, 2010). Attenuated activity of the insulin/IGF-1/PI3K pathway has been implicated in the development of metabolic diseases such as type-2 diabetes, and preservation of the activity of this signalling pathway is thought to be essential for glucose and lipid homeostasis in the long term (Engelman et al, 2006). This notion is challenged by the observation that loss-of-function mutations in components of the IIS pathway can reduce biomarkers of aging and extend lifespan in worms, flies and mice (Fontana et al, 2010; Kenyon, 2010).

Pharmacological and genetic approaches have revealed that, amongst the different PI3K family members, it is the p110α isoform of PI3K which selectively couples to insulin receptor substrate (IRS) proteins and plays the main role in insulin signalling (Foukas et al, 2006; Knight et al, 2006). We previously generated mice with heterozygous inactivation of p110α (p110α^D933A/WT^), by introducing a germline kinase-inactivating point mutation in *Pik3ca*, the gene encoding p110α (Foukas et al, 2006). The D933A mutation mimics systemic administration of p110α-selective small molecule inhibitors, but is embryonic lethal in homozygosity (Foukas et al, 2006). Heterozygous p110α^D933A/WT^ mice are viable, but exhibit hyperinsulinemia, glucose intolerance, hyperphagia and increased adiposity at a young age. However, these mice are not diabetic, as defined by hyperglycemia (Foukas et al, 2006). In line with the phenotype of p110α^D933A/WT^ mice, liver-specific deletion of p110α has similar adverse metabolic effects in young mice (Sopasakis et al, 2010).

The long-term effects of diminished p110α signalling are unknown. Gaining this information is important given that *PIK3CA*, the gene encoding human p110α, is frequently mutated in cancer and that clinical trials are under way to assess p110α as a therapeutic target in oncology (Vanhaesebroeck et al, 2010). The prospect of administration of p110α inhibitors to cancer patients has generated a strong interest in characterising the effects of long-term p110α inactivation on mammalian physiology and metabolism.

We report here that p110α^D933A/WT^ mice are resistant to age-related fat accumulation and that male mice exhibit better glucose homeostasis than wild-type (WT) littermates at middle age. This beneficial effect of p110α inactivation on metabolism correlates with a modest extension in life span. Thus, the metabolic impact of attenuated p110α activity is age-dependent, with long-term organismal adaptation to the adverse effects of p110α inactivation at young age.

## RESULTS

### Male p110α^D933A/WT^ mice display better glucose homeostasis compared to WT littermates at middle age

**Figure 1:**
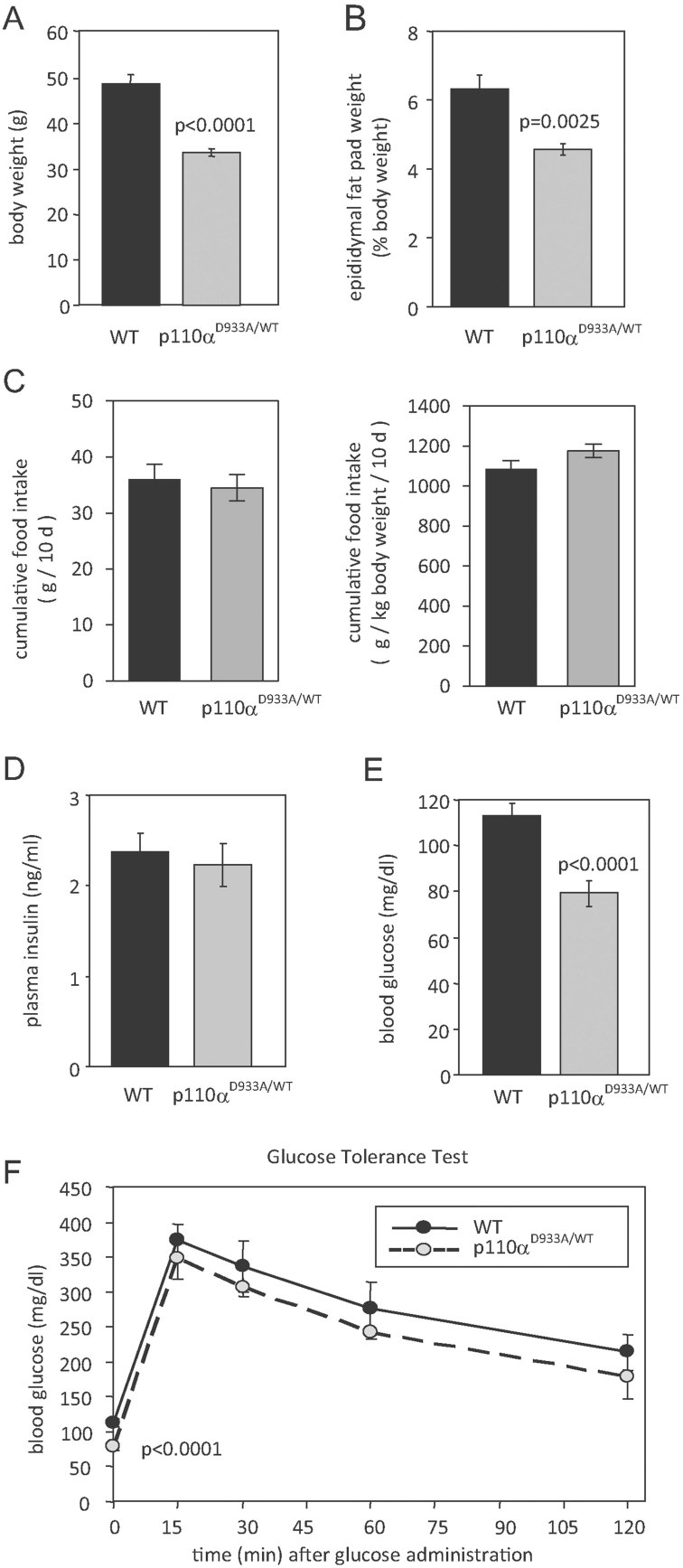
Middle-aged p110α^D933A/WT^ male mice are resistant to age-related fat accumulation and display better glucose homeostasis than WT littermates. Parameters were assessed in approximately 500-day-old male mice. Bars represent mean ± standard error. Statistical comparisons were performed with Student's *t*-test. A. Body weight (*n* = 17 per genotype). B. Epididymal fat pad weight (*n* = 3 per genotype). C. Cumulative food intake over 10 days (left panel), expressed per kg of body weight (right panel; *n* = 9 p110α^D933A/WT^–7 WT). D. Fasting plasma insulin (*n* = 5 per genotype). E. Fasting plasma glucose (*n* = 11 p110α^D933A/WT^–12 WT). F. Glucose tolerance injected intraperitoneally with 2 g glucose/kg body weight (*n* = 11 p110α^WT/D933A^–12 WT).

Mice heterozygous for the kinase-dead D933A allele of p110α (further referred to as p110α^D933A/WT^ mice) are hyperphagic and exhibit higher adiposity, insulin resistance and glucose intolerance at young age (Foukas et al, 2006). We expected such an adverse combination of metabolic parameters to predispose to further deterioration in overall fitness. Surprisingly, however, p110α^D933A/WT^ male mice were resistant to age-related fat accumulation. p110α^D933A/WT^ mice (male/female) are approximately 15% smaller than WT littermates at young age (approximately 50 days), but for the male mice this difference increased to approximately 30% at middle age (approximately 500 days; Fig 1A). p110α^D933A/WT^ mice displayed reduced adiposity at later ages as indicated by reduced weight of epididymal fat in the p110α^D933A/WT^ male mice (Fig 1B), despite the fact that their food intake was similar to that of WT littermates (Fig 1C). In line with reduced adiposity, middle-aged p110α^D933A/WT^ male mice displayed comparable insulin levels (Fig 1D), but lower fasting glucose levels (Fig 1E) and slightly better glucose tolerance (Fig 1F) compared with WT littermates. Female middle-aged p110α^D933A/WT^ mice were approximately 12% smaller than WT and exhibited nearly identical glucose homeostasis as WT littermates (Supporting Information Fig S1). This can be considered age-related improvement given that young female p110α^D933A/WT^ mice showed impaired glucose tolerance (Foukas et al, 2006).

**Figure 2:**
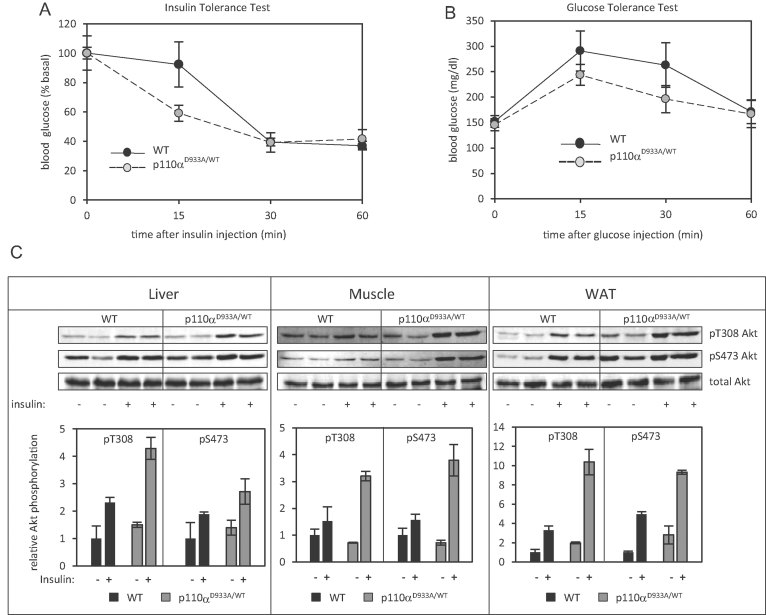
Middle-aged p110α^D933/WT^ mice fed a high fat diet maintain better metabolic profiles than WT littermates. Male mice were fed a high fat diet (45% calories derived from fat) at 66 weeks of age and for 14 weeks and were subsequently metabolically phenotyped as follows: A. Intraperitoneal insulin (2 U/kg) tolerance test (*n* = 6 per genotype). B. Intraperitoneal glucose (1 g/kg) tolerance tests (*n* = 6 per genotype). C. Insulin-stimulated Akt phosphorylation. Mice were injected with insulin (0.25 mU/g) via the *inferior vena cava* and Akt phosphorylation was assessed by quantitative immunoblot analysis in tissue homogenates [liver, *gastrocnemius* muscle, and white adipose tissue (WAT)]. Each lane represents an individual mouse (*n* = 4 per genotype). Signal intensity was quantified and data (mean ± standard deviation) are shown in the graphs below the respective blots.

We subjected male mice to high fat feeding (45% of calories derived from fat) starting at 66 weeks of age, for a period of 14 weeks. Weight gain in these middle-aged mice (Supporting Information Fig S2) was moderate and the rate comparable between p110α^D933A/WT^ and WT littermates. Nevertheless, high fat diet-fed p110α^D933A/WT^ mice maintained better glucose tolerance and insulin sensitivity than WT littermates, as assessed by glucose and insulin tolerance tests (Fig 2A and B). In terms of insulin signalling, Akt phosphorylation was higher in tissues of p110α^D933A/WT^ mice than in WT littermates following stimulation with insulin *in vivo* (Fig 2C). Class IA PI3K isoform expression, although somewhat variable, remained similar between aged WT and p110α^D933A/WT^ littermates (Supporting Information Fig S3). Therefore, the better insulin sensitivity in p110α^D933A/WT^ mice is not due to compensatory alterations in PI3K isoform expression.

### Chronic pharmacological inhibition of p110α protects from insulin resistance induced by sustained metabolic stress

Prolonged treatment of insulin-sensitive cells with insulin, saturated fatty acids or high concentrations of glucose suppresses insulin sensitivity, amongst other through down-regulation of the levels of insulin receptor substrate (IRS) proteins following their serine phosphorylation by kinases activated through negative feedback loops (Boura-Halfon & Zick, 2009). We modelled the effect of hyperinsulinemia, free fatty acid and hyperglycemia on insulin signalling in cell culture by prolonged (16 h) treatment of C2C12 mouse myotubes with insulin (10 nM), saturated fatty acid (palmitate, 500 µM) or high glucose (25 mM, instead of 5.5 mM).

**Figure 3:**
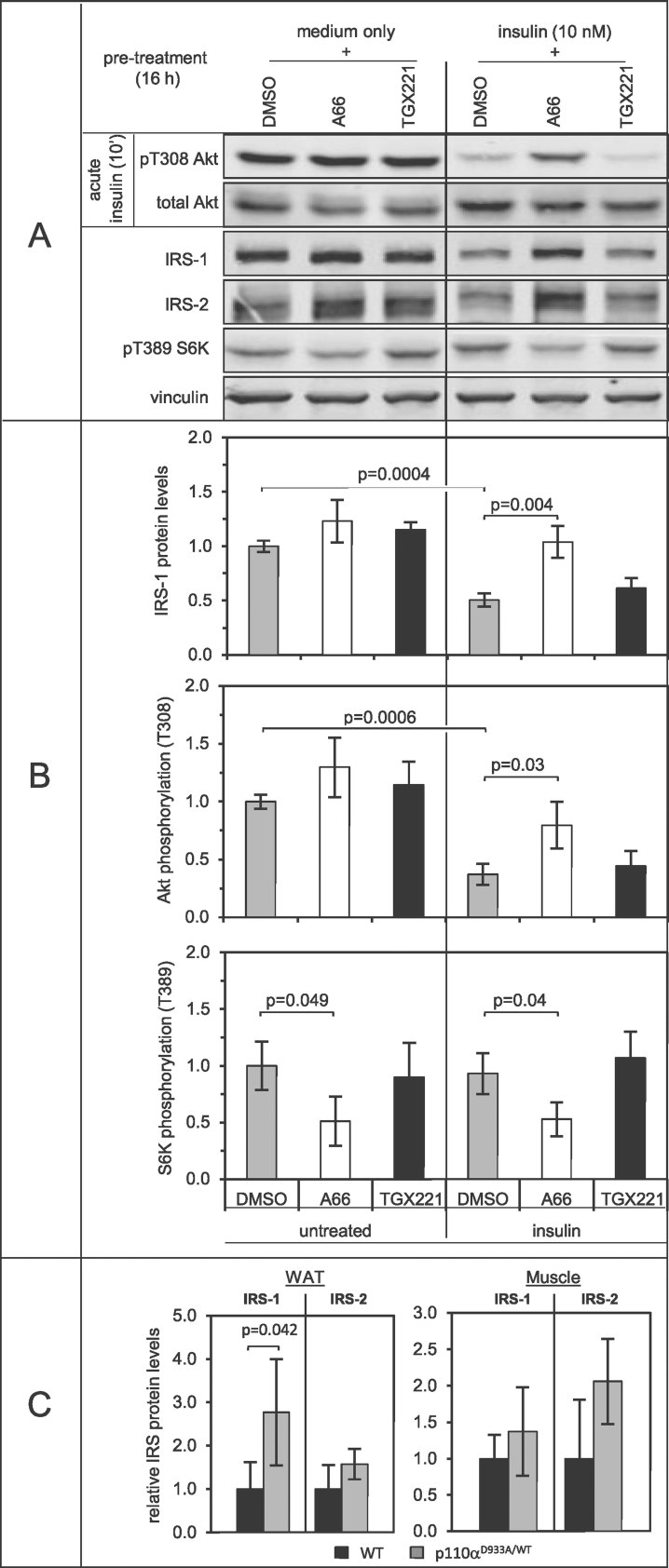
Chronic p110α inhibition promotes IRS-dependent signalling. A. p110α inhibition prevents IRS downregulation induced by chronic insulin treatment in C2C12 myotubes. C2C12 myotubes (cultured in medium with 5.5 mM glucose) were pre-treated for 16 h with insulin (10 nM) in the presence or absence of A66 (2 µM) or TGX-221 (1 µM). Monolayers were then rinsed with phosphate-buffered saline and lysed either directly for detection of IRS and S6K T389 phosphorylation or after 1 h culture in serum-free medium and re-stimulation with insulin (10 nM for 10 min; referred to in the figure as ‘acute insulin’) for detection of Akt T308 phosphorylation. Lysates were processed for immunoblot analysis with the indicated antibodies. Panels have been rearranged for clarity, but they are from the same immunoblots. Signal intensity was quantified and normalized to that of vinculin, used as a loading control in all immunoblots. B. Quantitative data (mean ± standard deviation) from three independent experiments performed as in A. Statistical comparisons were performed with Student's *t*-test. C. IRS protein levels (mean ± standard deviation) as determined by immunoblot analysis in epididymal fat pads [white adipose tissue (WAT)] and *gastrocnemius* muscle homogenates of 80 week old male p110α^D933A/WT^ mice and WT littermates (*n* = 4 per genotype).

Prolonged treatment with insulin resulted in down-regulation of IRS protein levels (Fig 3A and B). However, inclusion during treatment of the p110α-selective inhibitor (A66) (Jamieson et al, 2011), but not of the p110β-selective inhibitor TGX-221, protected IRS from depletion (Fig 3A and B). The protective effect of chronic p110α inhibition on IRS protein levels was reflected in improved insulin signalling output, as evidenced by increased Akt phosphorylation upon acute (10 min) stimulation with insulin (Fig 3A and B).

Albeit to a lesser extent than that caused by chronic insulin treatment, treatment with palmitate or high glucose also resulted in the downregulation of IRS, which could be prevented by inclusion of A66 (Supporting Information Fig S4A and B). In the presence of high glucose, IRS protein levels were also reduced, but this was not accompanied by reduced Akt phosphorylation (Supporting Information Fig S4A and B). Nevertheless, treatment with high glucose together with insulin and palmitate, a condition more relevant to actual metabolic pathologies, led to A66-enhanced Akt activation, compared to vehicle-treated cells (Supporting Information Fig S4C) and similar to that observed upon chronic treatment with insulin (Fig 3A and B). Similar results were obtained in 3T3-L1 adipocytes, in which the levels of IRS-2 were down-regulated upon sustained metabolic stimulation, albeit not as prominently as in myotubes (Supporting Information Fig S5).

A well-established negative feedback loop in insulin signalling is mediated by S6 kinase which phosphorylates IRS, leading to its degradation (Harrington et al, 2004; Shah et al, 2004). In both C2C12 myoblasts and 3T3-L1 adipocytes, prolonged treatment with the p110α-selective inhibitor A66 reduced phosphorylation of S6K on Thr389, a marker of activity of this kinase (Fig 3A and B and Supporting Information Fig S4).

Importantly, while young p110α^D933A/WT^ mice and WT littermates express similar levels of IRS-1 and -2 (Foukas et al, 2006), white adipose tissue and skeletal muscle of aged (80 week) p110α^D933A/WT^ male mice tended to contain higher levels of both IRS isoforms than WT littermates (Fig 3C and Supporting Information Fig S6), consistent with a protective effect of p110α inactivation on IRS downregulation induced by age-related hyperinsulinemia. Levels of activated S6K (assessed by Thr389 phosphorylation) in the same tissues tended to be somewhat reduced in p110α^D933A/WT^ mice (Supporting Information Fig S7).

Overall, these data demonstrate that chronic inactivation of p110α can have a positive effect on insulin signalling which could underlie, at least in part, the beneficial effects of p110α inactivation on glucose homeostasis. Chronic p110α inactivation might thus ameliorate the impact of age-related hyperinsulinemia and the lipotoxic effect of free fatty acids by promoting acute IRS-dependent insulin signalling.

### Partial inactivation of p110α results in a modest lifespan extension in male mice

**Figure 4:**
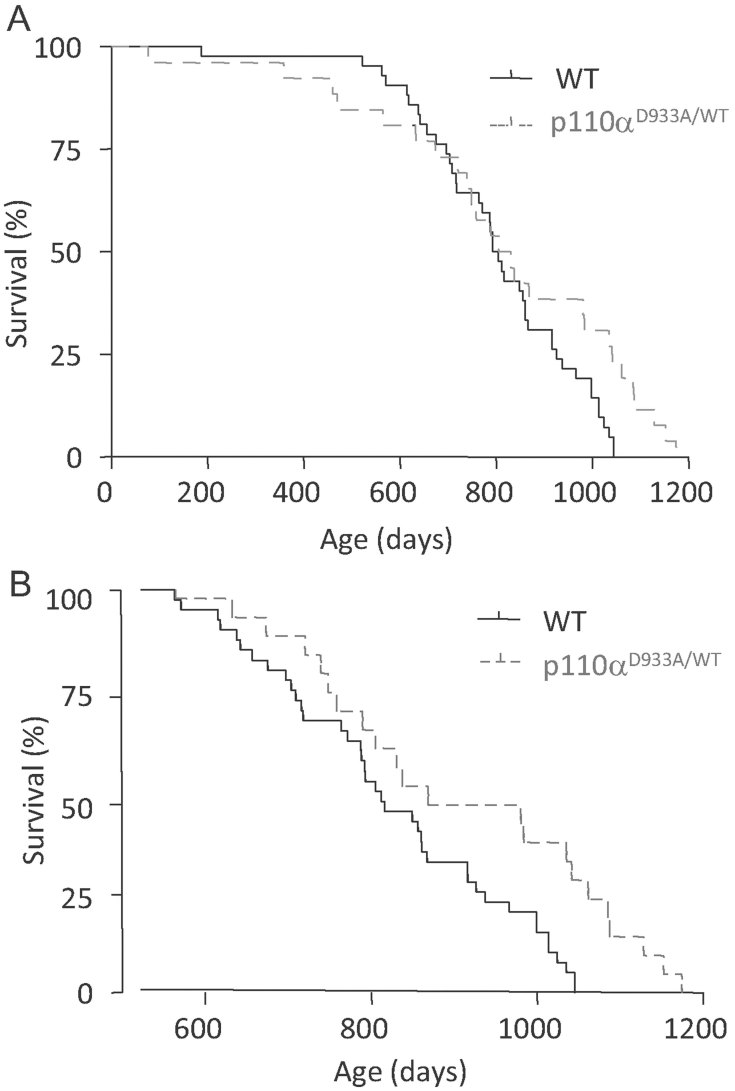
Extension of lifespan of male p110α^D933A/WT^ mice. A. Survival curves of p110α^D933A/WT^ mice. Cohorts of p110α^D933A/WT^ (*n* = 26) and WT (*n* = 42) male mice were monitored from birth to death as described in Materials and Methods. Median lifespan was 818 days (p110α^WT/D933A^) *versus* 799 days (WT). The difference between the curves is marginally statistically significant (log-rank test, *p* = 0.0545, *x*^2^ = 3.697). B. Survival analysis limited to mice surviving to 500 days of age (*n* = 21 for p110α^WT/D933A^ and *n* = 40 for WT). Median life span was 852 days (p110α^WT/D933A^) *versus* 805 days (WT). The difference between the curves is statistically significant (log-rank test, *p* = 0.0094, *x*^2^ = 6.739).

Inactivation of various components of the IIS pathway increases the life span of various organisms, including mice (Fontana et al, 2010; Kenyon, 2010). We therefore assessed whether inactivation of p110α would also affect lifespan in mice. We generated cohorts of male and female p110α^D933A/WT^ and WT littermate mice and maintained these under the conditions described in Materials and Methods. As shown in Fig 4A, male p110α^D933A/WT^ mice suffered increased mortality between approximately 50–500 days of age, presumably due to an as yet unidentified pathologic effect of p110α inactivation. Therefore, survival monitored from birth onwards showed a marginally statistically significant difference (*p* = 0.0545) in median life span of WT and p110α^D933A/WT^ male mice (799 days vs. 818 days). However, if only mice that survived to middle age (500 days) were taken into account, then male p110α^D933A/WT^ mice have a statistically significant median lifespan extension of approximately 6% (852 days vs. 805 days) compared with WT littermates (Fig 4B). This correlates with the improved glucose homeostasis in male p110α^D933A/WT^ mice. There was no difference in lifespan between female p110α^D933A/WT^ mice and WT littermates (Supporting Information Fig S1D, Tables S1 and S2), in line with the identical glucose homeostasis observed in female mutant and WT mice (Supporting Information Fig S1B and C).

Apart from the difference in body weight, the external appearance of p110α^D933A/WT^ mice did not differ from that of WT littermates at any age. Histopathological examination of a range of tissues (brain, skin, stomach, aorta, muscle, liver, kidney and lung) of approximately 500-day-old male mice did not reveal any substantial differences between WT and p110α^D933A/WT^ animals. Immunological markers on T cells, reported to correlate with longevity in mice, *i.e.* distribution of T cells in naive and memory subsets (Miller, 2001), were also unaffected (Supporting Information Fig S8). Taken together, these findings are in line with the modest magnitude of the lifespan extending effect of p110α inactivation.

## DISCUSSION

*Age-1*, the *Caenorhabditis elegans* ortholog of p110 PI3K, was the first IIS pathway component of which mutation was shown to extend lifespan over a decade ago (Morris et al, 1996), yet the impact on lifespan of inactivation of p110α, the principal insulin-responsive PI3K isoform in mammals, remains unknown. We have addressed this question using our previously characterised p110α^D933A/WT^ mutant mouse strain (Foukas et al, 2006). We found that these mice are resistant to age-related fat accumulation, when maintained on a standard diet. Consistent with this reduced adiposity, p110α^D933A/WT^ mice displayed better glucose homeostasis at middle age. This correlated with a modest median life span extension of approximately 6% in the male p110α^D933A/WT^ mice that survived to middle age.

The lifespan-extending effect of the p110α^D933A^ mutation is less than that observed in other IIS pathway mutant mice. For instance, female IRS-1 and S6K1 knock-out mice, have a 10% and 19% median lifespan expansion, respectively (Selman et al, 2008, 2009), but it should be kept in mind that these mice are homozygous for the mutant allele. Interestingly, while mutations in the IIS pathway have been shown to greatly extend the lifespan of flies (Clancy et al, 2001; Tatar et al, 2001), inducible overexpression of a dominant-negative Dp110 (the fly ortholog of p110α) in female adult flies has recently been reported to also result in only a modest (7%) median lifespan extension (Slack et al, 2011), comparable with that seen in male p110α^D933A/WT^ mice in this study. Furthermore, a recent study that re-assessed the lifespan of heterozygous IGF-1 receptor (IGF-1R) mice found only a modest 5% extension in the mean lifespan of female mice (Bokov et al, 2011), which is also comparable with that seen in male p110α^D933A/WT^ mice. Circulating IGF-1 levels are reduced in long-lived hypopituitary dwarf mice and growth hormone receptor (GHR) knockout mutants, but the modest lifespan extending effect of IGF-1R mutation suggests that the longevity of these mutants is not related to reduced IGF-1 levels in these mutants (Brown-Borg & Bartke, 2012). Along these lines, circulating IGF-1 levels in young at least p110α mice are not significantly different from those in WT littermates (Supporting Information Fig S9). Of note, IGF-1 levels are not reduced in long-lived S6K1 mutant mice (Selman et al, 2009), which shows that reduced levels of IGF-1 is not a common feature of all long-lived mouse mutants.

Another point to note is that lifespan extension in mouse knockouts of IGF-1R, or S6K1 has only been found in female mice or in the case of IRS1 is more pronounced in female null mice (Selman et al, 2011). This is not the case in p110α^D933A/WT^ mice in which male mice had the beneficial metabolic phenotypes in old age and an associated increase in lifespan. This is an important point because evolutionary theories of ageing predict plasticity in the life span of females as a result of trade-offs between longevity and fecundity (Partridge et al, 2005). Females with mutations in the insulin signalling pathway often show reduced fecundity that could explain their increased lifespan. The present study shows that insulin pathway attenuation can also extend lifespan in males, presumably, through mechanisms not related to fecundity.

IIS diverges into two branches downstream of IRS-1, one towards PI3K/Akt and the other to Grb2/Ras/ERK. This means that inactivation of p110α only partially recapitulates the impact of IRS-1 deletion. It is possible that independent or concomitant down-regulation of Grb2/ERK signalling is required in order to achieve a greater increase in lifespan. It is also possible that p110α, but not IRS, operates in signalling pathways whose down-regulation might have adverse health effects. For instance, mice with cardiac-specific inactivation of p110α suffer from cardiac dysfunction (Lu et al, 2009; Pretorius et al, 2009), collateral adverse effects that might limit the overall life-extending effect of attenuation of p110α activity. Notably, however, suppression of p110α has been reported to impair the cardiac function of young mice, but to preserve that of old mice likely by delaying senescence in cardiac tissue (Inuzuka et al, 2009).

As the PI3K pathway is implicated in numerous cellular processes, the question of the mechanism underlying lifespan extension in mice has no straightforward answer. For instance, the PI3K pathway regulates autophagy, a cellular process implicated in aging and life span extension in insulin pathway mutants (Cuervo, 2008). Although we cannot exclude that autophagic activity in adult mouse organs could be altered as a result of p110α inactivation, a detailed assessment of autophagy in mouse embryonic fibroblasts derived from p110α^D933A/WT^ mice did not reveal any significant impact of partial p110α inactivation on autophagy (Supporting Information Fig S10). The possible impact of p110α inactivation on the expression/activity of FOXO transcription factors, known to have broad metabolic regulatory functions (Accili & Arden, 2004), would also merit further investigation. FOXOs are also known to regulate cellular antioxidant capacity, but recently, the causal implication of oxidative stress to the ageing process has become more controversial (Gems & Partridge, 2013).

**Figure 5:**
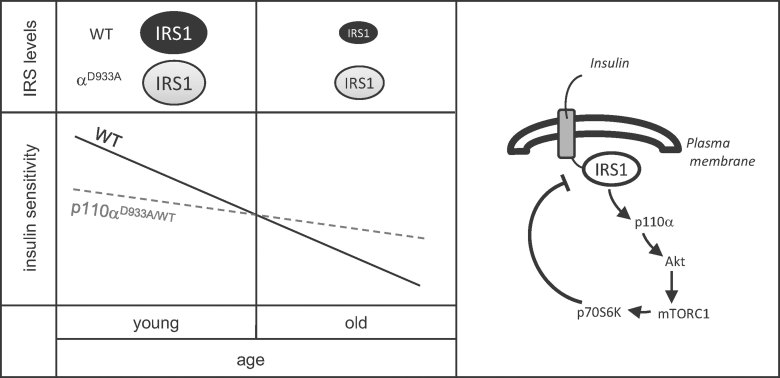
Schematic overview of the beneficial impact of chronic p110α inactivation on age-related deterioration of insulin sensitivity. The levels of IRS are key to insulin signalling. Chronic stimulation with insulin, as occurs in hyperinsulinemia, downregulates IRS via a negative feedback loop that involves IRS phosphorylation by S6K, exacerbating age-related insulin resistance. Reduced p110α activity as a consequence of loss-of-function mutation (D933A) or pharmacologic inhibition (*e.g.* A66) attenuates S6K activity, protecting against chronic insulin-induced IRS depletion, thus promoting insulin sensitivity.

On the other hand, it is plausible that the beneficial metabolic effect of partial p110α inactivation observed in aged mice could underlie lifespan extension. Aged p110α^D933A/WT^ mice are leaner than WT littermates and this is a common feature of most mice with mutations in the insulin pathway that provide extended lifespan. Along the same lines, the apparent positive impact of long-term p110α inactivation on insulin signalling could be due to lower susceptibility to lipotoxic effects of age-related obesity. Our data from prolonged pharmacological inhibition of p110α in cell lines and from studies in aged p110α^D933A/WT^ mice suggest that attenuated p110α activity could ameliorate the impact of age-related hyperinsulinemia and the lipotoxic effect of free fatty acids by promoting acute IRS-dependent insulin signalling. These data provide a potential mechanism to explain improved glucose homeostasis in aged p110α^D933A/WT^ male mice compared to their wild-type littermates (shown schematically in Fig 5). However it should be mentioned that reduced adiposity is not a universal feature of all long-lived mutants. For instance, GHR knockout mice are long living despite having higher adiposity (Bonkowski et al, 2006). Visceral fat is thought to impair insulin sensitivity and negatively impact on metabolic homeostasis. Hence, surgical removal of visceral fat provided the expected positive effect on the insulin sensitivity of WT mice but, surprisingly, had a negative impact on the insulin sensitivity of GHR knockout mice (Masternak et al, 2012). This shows that GHR mutation profoundly alters the biology of visceral adipose tissue in a beneficial way for metabolic homeostasis (Masternak et al, 2012). For instance, GHR knockout mice have reduced levels of pro-inflammatory cytokines implicated in development of insulin resistance (TNF-α and IL-6), possibly as a consequence of anti-inflammatory effects of increased levels of adiponectin in these mutants (Masternak & Bartke, 2012). In the case of p110α^D933A/WT^ mice, adiponectin levels are significantly elevated in young mice (Dubois et al, 2012), but in the face of coexistent insulin resistance, this likely reflects adiponectin resistance, rather than indicating a beneficial action of adiponectin related to the observed lifespan extension. It is possible that inactivation of p110α has similar effects as GHR knockout on adipose tissue biology and the inflammatory profile of mutant mice. Adipose tissue-specific p110α mutagenesis experiments aiming to address these possibilities are under way in our lab.

**The paper explained**

**PROBLEM**

The gene encoding the p110α PI 3-kinase (PI3K) is one of the most frequently mutated genes in human cancer. This kinase is therefore actively pursued as a therapeutic target in oncology. p110α is known to be important in insulin signalling and glucose homeostasis. Indeed, partial inactivation of p110α in young mice leads to insulin resistance and glucose intolerance. Similarly, short-term administration of p110α inhibitors in clinical trials in human cancer, also induces acute metabolic side-effects. It is unknown if long-term treatment with p110α inhibitors, as might be needed in cancer, will lead to diabetes or other metabolic disturbances. It is therefore important to understand the impact of sustained inactivation of p110α on the organism. The PI3K pathway has also been implicated in organismal ageing.

**RESULTS**

Using a genetic mouse model that mirrors partial pharmacological inactivation of p110α, we found that sustained p110α attenuation protects from fat accumulation and deterioration in insulin sensitivity upon ageing and leads to better overall glucose homeostasis compared with control aged mice. The beneficial effect of p110α inactivation is partially due to preservation of insulin/IRS signalling complexes, attenuating the age-related decline of insulin signalling. Consistent with this, mice with partial inactivation of p110α have a modest life span extension.

**IMPACT**

This is an example of an undesirable short-term side-effect of kinase inactivation that leads to organismal adaptation, with unanticipated beneficial effects in the long-term. p110α inhibitors are a potential new therapy for cancer. The side effects of these drugs on glucose metabolism are a concern. This study provides evidence that sustained therapeutic inactivation of p110α might not have a detrimental impact on the glucose homeostasis of patients.

The precise mechanism underlying leanness in aged p110α^D933A/WT^ mice remains to be defined. To this end, further characterisation of metabolic physiology of aged p110α^D933A/WT^ mice by indirect calorimetry, core body temperature and spontaneous motor activity measurements should provide additional information. However, such parameters do not necessarily correlate with either adiposity or extended longevity. For instance, S6K1 knockout mice have reduced adiposity and extended lifespan, but no difference in core body temperature, thyroid-stimulating hormone levels or resting metabolic rates compared to WT littermates in contrast to hypopituitary dwarf and GHR knockout mice (Selman et al, 2009). Previous *ex vivo* studies have indicated that p110α is required for adipogenesis (Kim et al, 2009; Zhao et al, 2006). However, the apparent increased adiposity of young p110α^D933A/WT^ mice suggests that the resistance to age-related fat accumulation is unlikely to be a manifestation of impaired development of the adipose tissue. A potential mechanism for the reduced fat mass could be due to alterations in the signalling pathways in the central nervous system that regulate energy homeostasis. However, we have recently reported that inactivation of p110α specifically in POMC or AgRP hypothalamic neurons has no substantial effect on food intake or body weight and adiposity of mice, as these physiological processes are largely mediated by p110β (Al-Qassab et al, 2009). Therefore, the mechanism underlying resistance in fat accumulation in aged p110α^D933A/WT^ mice more likely involves altered energy metabolism. Such a mechanism was also observed upon transgenic overexpression in mice of PTEN (Garcia-Cao et al, 2012; Ortega-Molina et al, 2012), a phosphatase which dephosphorylates the lipids produced by the class I PI3K isoforms (p110α, p110β, p110γ and p110δ). These mice exhibit enhanced oxidative metabolism and improved energy and glucose homeostasis (Garcia-Cao et al, 2012; Ortega-Molina et al, 2012). Transgenic PTEN overexpression models the inactivation of all class I PI3K isoforms and might also affect PI3K-independent signalling pathways, due to lipid phosphatase-independent roles of PTEN (Song et al, 2012). This contrasts with our studies which uncover the selective impact of partial inactivation of p110α, and also model the action of p110α-selective small molecule inhibitors.

Taken together, our findings show that p110α inactivation has an age-dependent beneficial effect on metabolism. This suggests that long-term inhibition of p110α in the clinic might not have a detrimental effect on glucose metabolism, as would be expected based on the impact of p110α inactivation in young mice (Foukas et al, 2006) and the negative impact of acute class I PI3K inhibition in human cancer patients in phase I/II trials (Bendell et al, 2012).

## MATERIALS AND METHODS

### Mouse maintenance and lifespan determination

p110α^D933A/WT^ mice have been described earlier (Foukas et al, 2006). Mice in this study had been backcrossed to C57BL/6 background for 11 generations. Cohorts of mice were generated by crossing female WT mice with male p110α^D933A/WT^ mice. Mice recruited in this study were born within a period of less than 3 months. Mice were housed in individually ventilated cages with *ad libitum* access to water and standard chow (RM I, Special Diet Services) or high fat diet (45% calories from fat, cat no. 824053, Special Diet Services). Health status was monitored daily and body weight was recorded periodically. Moribund mice were euthanized and subjected to an autopsy. All experimental procedures complied with the UK Home Office Animals (Scientific Procedures) Act 1986 and were performed in accordance with local Ethical Committee guidelines.

### Metabolic analysis

Metabolic analysis was performed as previously described (Foukas et al, 2006). Aged mice fed a high fat diet were subjected to glucose and insulin tolerance tests by intraperitoneal injection of glucose (1 g/kg) and insulin (2 U/kg, Actrapid, Novo Nordisk), respectively. Insulin signalling was assessed in tissues (liver, *gastrocnemius*, epididymal fat pad) dissected 10 min following injection of a bolus of insulin (0.25 mU/g of body weight) through the inferior *vena cava* of terminally anaesthetized mice. Quantitative immunoblot analysis was performed using an Odyssey infrared scanner and software (LiCOR).

### Cell culture and treatments

3T3-L1 cells were cultured and differentiated to adipocytes according to (Zebisch et al, 2012). Differentiated adipocytes were switched to DMEM with 5.5 mM glucose (low glucose medium) 24 h before initiation of treatments. C2C12 myoblasts were cultured in DMEM with 5.5 mM glucose, supplemented with 10% foetal bovine serum, until they reached confluency, then transferred in 2% horse serum containing medium, which was replaced daily over 4 days at which point they formed myotubes. Palmitate (Sigma) was dissolved in ethanol, diluted at 1 mM in DMEM with 10% FBS, sonicated and filter sterilized.

### Statistical analysis

Statistical comparisons were performed by Student's *t*-test. Survival was analysed by Kaplan–Meier survival curves and differences were assessed for statistical significance by log-rank test. The number of animals in each group is indicated by *n*. Statistical analyses were performed using GraphPad Prism software.

For more detailed Materials and Methods see the Supporting Information.

## Author contributions

LCF, DJW and BV designed research; LCF, BB, LB, WP, KA and SS performed research; all authors analysed data; LCF and BV wrote the paper.

## For more information

OMIM database entry:

http://www.omim.org/entry/171834?search=pik3ca&highlight=pik3ca

## Notes

As a service to our authors and readers, this journal provides supporting information supplied by the authors. Such materials are peer reviewed and may be re-organized for online delivery, but are not copy-edited or typeset. Technical support issues arising from supporting information (other than missing files) should be addressed to the authors.
