# Supplementary material for: Long-term p110α PI3K inactivation exerts a beneficial effect on metabolism
Source: EMBO Mol Med. 2013 Mar 11;5(4):563–71. doi: 10.1002/emmm.201201953 (PMC3628103; doi:10.1002/emmm.201201953)
Supplement: Supplementary file 1 [file emmm0005-0563-sd1.pdf]

EMBO Molecular Medicine

Long-term p110 PI3K inactivation exerts a beneficial effect on metabolism

Lazaros C. Foukas, Benoit Bilanges, Lucia Bettedi, Wayne Pearce, Khaled Ali, Sara Sancho, Dominic J. Withers and Bart Vanhaesebroeck

Corresponding author: Lazaros Foukas, University College London

Review timeline:

Submission date:	30 August 2012
Editorial Decision:	04 October 2012
Additional Author Correspondence:	05 October 2012
Editorial Decision:	16 October 2012
Additional Author Correspondence:	18 October 2012
Editorial Decision:	28 November 2012
Revision received:	03 January 2013
Accepted:	07 January 2013

Transaction Report:

(Note: With the exception of the correction of typographical or spelling errors that could be a source of ambiguity, letters and reports are not edited. The original formatting of letters and referee reports may not be reflected in this compilation.)

Editors: Anneke Funk / Natascha Bushati

1st Editorial Decision 04 October 2012

Thank you for the submission of your manuscript "Long-term p110 PI3K inactivation exerts a beneficial effect on metabolism" to EMBO Molecular Medicine. We have now heard back from the three referees whom we asked to evaluate your manuscript. You will see that they find the topic of your manuscript potentially interesting. However, they also raise some concerns on the study, which should be addressed in a revision of the manuscript.

Importantly, reviewer #2 highlights that the animals should be characterized in more detail regarding several factors like serum adipokine concentrations and compensatory adaptations.

Given the balance of these evaluations, we feel that we can consider a revision of your manuscript if you can convincingly address the issues that have been raised within the time constraints outlined below.

Revised manuscripts should be submitted within three months of a request for revision. They will otherwise be treated as new submissions, unless arranged otherwise with the editor.

I look forward to seeing a revised form of your manuscript as soon as possible.

© EMBO

***** Reviewer's comments *****

Referee #1:

I have reviewed the paper from Withers and Vanhaesebroeck and find it acceptable for publication. Both the senior authors are experts in this field of signaling and have made extensive contributions. This short manuscript is a continuation of their ongoing studies.

Referee #2 (Comments on Novelty/Model System):

The manuscript by Foukas et al presents information about the long-term effects of p110 inactivation on metabolism and lifespan in mice. The authors show that chronic p110 inactivation in mice protects from age-related reduction in insulin sensitivity, glucose tolerance and fat accumulation, and extends the lifespan of male mice. This is a very interesting set of data that further highlights the importance of the insulin/IGF pathway in regulating metabolism and aging in mammals.

Referee #2 (Other Remarks):

The manuscript by Foukas et al presents information about the long-term effects of p110 inactivation on metabolism and lifespan in mice. The authors show that chronic p110 inactivation in mice protects from age-related reduction in insulin sensitivity, glucose tolerance and fat accumulation, and extends the lifespan of male mice. This is a very interesting set of data that further highlights the importance of the insulin/IGF pathway in regulating metabolism and aging in mammals.

Comments:
1. Did the authors measure mesenteric fat ? Any data on fat free mass and bone mass ? It would be important to show these data in the manuscript.
2. It would be important to know if there are differences in serum adipokine (e.g. leptin, adiponectin, IL6, TNF) concentrations between the p110 and WT mice.
3. In several dietary and genetic animal models of longevity there are similar hormonal (i.e. reduction in IGF-1, thyroid hormones) and metabolic (e.g. reduction in core body temperature) compensatory adaptations. It would be interesting to see some of these measurements reported in this manuscript.
4. It would be important to have more molecular data of the downstream pathways related with longevity in liver, adipose tissue and skeletal muscle. Is FOXO up-regulated ? What about the mTOR down-stream targets ?
5. More detailed information should be provided regarding food intake measurement. Did the author measure spontaneous motor activity ?

Additional Author Correspondence 05 October 2012

Thank you for considering our manuscript on p110alpha PI3K/mouse ageing for publication in EMM, we are very pleased that both Referees are positive about our findings.

In response to Referee 2, who commented that 'this is a very interesting set of data' and also suggested expanding on the phenotypic characterisation of these mice, we agree that such additional characterisation would be of interest. However, most of these phenotypes are rather predictable, as they have been documented before in other insulin signalling pathway mutants. We therefore feel that the requested experiments are unlikely to make a significant addition to the main findings of our paper, both conceptually and with respect to implications for therapeutic targeting. Importantly, addressing these comments would also take a very substantial amount of time and investment as detailed below.

In summary, in its current version, our manuscript convincingly documents novel, unexpected and therapeutically relevant phenotypes of p110a PI3K inactivation. We believe that adding further details will not significantly enhance the message of our paper and we hope you will find it suitable for publication in EMM.

Detailed comments:

At present, we do not have aged mice available. In order to address the comments below, we estimate it would take a cohort of around 10-15 mice per genotype and 14-17 months of breeding and ageing to >1 year, before subjecting the mice to metabolic phenotyping.

1. Did the authors measure mesenteric fat? Any data on fat free mass and bone mass? It would be important to show these data in the manuscript.

We have not measured mesenteric fat, but we believe that the epididymal fat pad weight which we have reported provides a good and widely accepted measure of adiposity. Similarly, we have not measured overall adiposity which would provide a measure of lean mass or the bone density. We have previously performed these measurements in young mice (Foukas et al 2006, Nature 441:366), and although it would be interesting to see if these parameters changes upon ageing, they are probably not essential descriptors of the phenotypes reported in our manuscript.

2. It would be important to know if there are differences in serum adipokine (e.g. leptin, adiponectin, IL6, TNFa) concentrations between the p110alpha; and WT mice.

Again we have not performed such measurements in aged mice. Leptin and adiponectin levels in young p110a kinase-dead mice are significantly higher than in WT mice. Such measurements are frequently reported in ageing/metabolic studies, however in our view, the interpretation of such measurements is not straightforward. For instance, higher levels could result from higher adiposity, leptin-adiponectin-resistance or other factors, which are usually not investigated given that this would require substantial experimentation.

3. In several dietary and genetic animal models of longevity there are similar hormonal (i.e. reduction in IGF-1, thyroid hormones) and metabolic (e.g. reduction in core body temperature) compensatory adaptations. It would be interesting to see some of these measurements reported in this manuscript.

Indeed, it would be interesting to see these parameters with reference to those reported for other mutants in the pathway. However, there is not always a clear correlation of such parameters with longevity or metabolic phenotypes. For instance, in mice deficient for IRS-1 (an upstream regulator of p110a), there is no significant difference in IGF-1 levels, despite extended longevity and IGF-1 resistance.

4. It would be important to have more molecular data of the downstream pathways related with longevity in liver, adipose tissue and skeletal muscle. Is FOXO up-regulated ? What about the mTOR down-stream targets?

We have already looked at insulin signalling in those tissues and found that p110a mutant mice remain more insulin sensitive as assessed by Akt phosphorylation. However, the activation status of the Foxo and mTOR pathway would be dependent on post-translational modifications such as their phosphorylation and importantly in the case of Foxo acetylation. These studies are not straightforward in mouse tissues. In our experience, there is a lot of variation amongst individual mice and obtaining reliable data would require very high numbers of mice. Literature data on the activity of these pathways in tissues are most often not convincing.

5. More detailed information should be provided regarding food intake measurement. Did the author measure spontaneous motor activity?

We have performed precise food intake measurements according to standard protocols (as reported in Fig 1C).

The spontaneous motor activity has not been assessed, but it is certainly an important parameter that we would like to investigate in future studies.

Editorial Decision 16 October 2012

Thank you for your message and please accept my apologies for the delayed reply.

I now had the opportunity to carefully re-read your manuscript and considered your letter, your answers to the reviewer's comments as well as the related literature again. I have also discussed them with my colleagues and consulted with our Chief Editor. I am afraid that we are not convinced that the manuscript is well suited for publication in EMBO Molecular Medicine in absence of the additional complementary data asked for by the reviewer.

We appreciate your responses to the reviewer's comments and certainly acknowledge that the breeding and aging of the mice would be very time consuming. However, in our opinion, the data asked for by the reviewer are important to support the study especially since a conclusive mechanistic explanation for the observed effects remains to be shown. Hence, I am afraid that we cannot offer publication of the manuscript in EMBO Molecular Medicine without the mentioned data.

I am sorry to have to disappoint you at this stage.

Additional Author Correspondence 18 October 2012

We are wondering whether there would be the option to consult the Referee, to assess what she/he thinks of our rebuttal. Given the difficult situation we're in, this might not be an unreasonable thing to do.

We would be prepared to initiate the breeding for the requested experiments, and get back to EMBO Molecular Medicine in 12-24 months as long as the Referee still agrees that these experiments are indeed critical, once he/she has taken into account our feedback in our rebuttal.

Editorial Decision 28 November 2012

Thank you for your message asking us to reconsider our decision and please accept my apologies for not replying earlier. As Dr. Funk is currently away on maternity leave, your manuscript has been assigned to me.

I have carefully re-read your manuscript and considered your letter, your point-by-point response, as well as the related literature again. I have also discussed them with my colleagues and consulted with reviewer #2, an Advisory Board Member and our Chief Editor.

We agree with referee #2 that it would be ideal to perform the suggested experiments. However, we acknowledge that the novelty of the presented findings would be compromised if obtaining this data would take one year or longer.

Therefore, should you be able to provide the requested data, we would encourage you to include it into the present study. Otherwise, we would request from you to include an in-depth discussion of the limitations raised by reviewer #2 into the revised manuscript.

Please include a point-by-point response highlighting how you have addressed the issues raised when you submit your revised manuscript.

© EMBO

I look forward to reading a new revised version of your manuscript in due course.

1st Revision - authors' response 03 January 2013

Referee #1:

I have reviewed the paper from Withers and Vanhaesebroeck and find it acceptable for publication. Both the senior authors are experts in this field of signalling and have made extensive contributions. This short manuscript is a continuation of their on-going studies.

We thank the Referee for his/her favourable evaluation of our manuscript.

Referee #2:

The manuscript by Foukas et al. presents information about the long-term effects of p110a inactivation on metabolism and lifespan in mice. The authors show that chronic p110a inactivation in mice protects from age-related reduction in insulin sensitivity, glucose tolerance and fat accumulation, and extends the lifespan of male mice. This is a very interesting set of data that further highlights the importance of the insulin/IGF pathway in regulating metabolism and aging in mammals.

Comments:

1. Did the authors measure mesenteric fat? Any data on fat free mass and bone mass? It would be important to show these data in the manuscript.

We have not measured mesenteric fat, but we believe that the epididymal fat pad weight, which we show in the manuscript, provides a good and widely accepted measure of adiposity. Similarly, we have not measured overall adiposity, lean mass or bone density in old mice. We have previously performed such measurements in young mice (Foukas et al 2006, Nature 441:366). In the discussion of the revised manuscript, we acknowledge the importance of such measurements to allow for a comparison with other long-lived mouse mutants, notably with Growth Hormone pathway mutant mice in which visceral fat mass is higher but the biology of its constituent adipocytes is altered in a beneficial way as a result of the mutation. We also mention that experiments utilising tissue-specific p110a inactivation are under way in our laboratory aimed to address whether similar effects on the biology of adipocytes occur as a result of inactivation of p110a.

2. It would be important to know if there are differences in serum adipokine (e.g. leptin, adiponectin, IL6, TNFa) concentrations between the p110alpha and WT mice.

Such measurements are frequently reported in ageing/metabolic studies, however in our view, the interpretation of such measurements is not always straightforward. For instance, higher levels of leptin and/or adiponectin could result from higher adiposity, resistance to leptin and/or adiponectin, or other factors, which are usually not investigated in detail, given that this would require substantial experimentation. Leptin and adiponectin levels in young p110a$^{WT/D933A}$ mice (which are insulin-resistant and hyper-insulinemic) are significantly higher than in WT mice (Foukas et al 2006, Nature 441: 366; Dubois et al 2012, Cell Signal. 24: 1971). As now discussed in the revised manuscript, in the face of insulin resistance, such high levels of leptin and adiponectin likely reflect leptin- and adiponectin-resistance in these young p110a$^{WT/D933A}$ mice. Pro-inflammatory cytokine profiles are certainly important descriptors of metabolic phenotypes and although we have not performed such measurements in the current study, such investigations will be carried out in tissue-specific p110a mutant mice that are under development in our laboratory.

3. In several dietary and genetic animal models of longevity there are similar hormonal (i.e. reduction in IGF-1, thyroid hormones) and metabolic (e.g. reduction in core body temperature)

compensatory adaptations. It would be interesting to see some of these measurements reported in this manuscript.

Indeed, it would be interesting to compare these parameters in p110a$^{WT/D933A}$ mice with those of other long-lived mutants. Plasma IGF-1 levels are not different between WT and p110a kinase-dead young mice (new Fig. S10 in the revised manuscript). However, as now mentioned in the discussion of the revised manuscript, there is not always a clear correlation of such parameters either with longevity or with metabolic phenotypes. Recently, it has also become clear that IGF-1 Receptor mutant mice are not necessarily as long-lived as was initially reported (Bokov et al 2011, PloS one 6: e2689). Also, there are no significant differences in IGF-1 levels in mice deficient for IRS-1 (an upstream regulator of p110a), despite extended longevity and IGF-1 resistance in these mice. Similarly, long-lived S6K mutant mice show no significant differences in plasma levels of either IGF-1 or thyroid stimulating hormone or in core body temperature. These comments have now been inserted in the discussion of the revised manuscript.

4. It would be important to have more molecular data of the downstream pathways related with longevity in liver, adipose tissue and skeletal muscle. Is FOXO up- regulated? What about the mTOR down-stream targets?

We have assessed insulin signalling in metabolic tissues and found that aged p110a mutant mice tend to be more insulin-sensitive, as assessed by Akt phosphorylation. The same mice tend to have reduced mTOR signalling as evidenced by reduced S6K phosphorylation (new Fig. S7 in the revised manuscript). We do not have data on FOXO activity. A simple determination of FOXO expression levels would not necessarily be sufficient as their activity is regulated by post-translational modifications such as phosphorylation and acetylation. As we now acknowledge in the revised manuscript, FOXOs are likely to be involved in the reported metabolic phenotypes for p110a mutant mice. We intend to assess FOXO activity in a comprehensive analysis using a substantial number of mice in order to generate meaningful results in future studies. Indeed, in our own experience, there tends to be a lot of variation in FOXO expression levels and post-translational modification in individual wild-type mice and for this reason, obtaining reliable data will require large numbers of mice.

5. More detailed information should be provided regarding food intake measurement. Did the author measure spontaneous motor activity?

We have performed precise food intake measurements (presented in Fig 1C), according to standard protocols. The spontaneous motor activity has not been assessed in the present study, but as we now acknowledge in the discussion of our revised manuscript, this is certainly an important parameter that we would like to investigate in future studies.
