# Supplementary material for: Long-term p110α PI3K inactivation exerts a beneficial effect on metabolism
Source: EMBO Mol Med. 2013 Mar 11;5(4):563–71. doi: 10.1002/emmm.201201953 (PMC3628103; doi:10.1002/emmm.201201953)
Supplement: Supplementary file 2 [file emmm0005-0563-sd2.pdf]

Supporting Information

Long-term p110α PI3K inactivation exerts a beneficial effect on organismal metabolism in mice

Lazaros C. Foukas, Benoit Bilanges, Lucia Bettedi, Wayne Pearce, Khaled Ali, Sara Sancho, Dominic J. Withers and Bart Vanhaesebroeck

<u>Table of contents:</u>

Supplemental Materials and Methods

- GFP-LC3 mice.
- Immunoblotting and immunofluorescence assessment of autophagic activity.
- T cell surface marker expression.

Supplemental References

Figure S1 Glucose homeostasis (at middle age) and life span in p110$\alpha^{D933A/WT}$ female mice.

Figure S2 Weight gain in mice fed a high fat diet.

Figure S3 Class IA PI3K isoform expression in tissues from aged p110α^{D933A} mice.

Figure S4 Effect of chronic p110α inhibition on free fatty acid- and high glucose-induced IRS downregulation in C2C12 myotubes.

Figure S5 Effect of chronic p110α inhibition on IRS-dependent signaling in 3T3-L1 adipocytes.

Figure S6 IRS-1 and -2 protein levels in white adipose tissue (WAT) and skeletal muscle of aged mice.

Figure S7 Insulin stimulated S6K Thr389 phosphorylation in white adipose tissue (WAT) and skeletal muscle of aged mice.

Figure S8 Distribution of T cells in naive and memory cell subsets between WT and p110α^{D933A} mice.

Figure S9 Fasting plasma IGF-1 in young p110$\alpha^{D933A/WT}$ and WT littermate mice

Figure S10 Assessment of autophagy in MEFs derived from p110$\alpha^{D933A/WT}$ embryos.

Table S1 Survival characteristics of p110$\alpha^{D933A/WT}$ mice.

Table S2 Log-rank test comparisons and hazard ratios of p110$\alpha^{WT/D933A}$ mice.

Supplemental Materials and Methods

GFP-LC3 mice. Transgenic GFP-LC3 mice (1) were maintained on a C57BL/6 background and crossed with p110α$^{D933A/WT}$ mice to generate GFP-LC3/p110α$^{D933A/WT}$ mice. Mouse embryonic fibroblasts (MEFs) were isolated from GFP-LC3/WT and GFP-LC3/p110α$^{D933A/WT}$ intercrosses as described before (2). Genotyping was performed as previously described (1, 2).

Immunoblotting and immunofluorescence assessment of autophagic activity. For starvation experiments, cells were washed three times with Earle's Balanced Salt Solution (EBSS, starvation medium) and incubated in EBSS medium for the indicated time in the presence or absence of 50 nM of Bafilomycin A1 (Baf A1). For GFP-LC3 lipidation assays, cells were lysed in ice-cold lysis buffer containing 1% NP-40, 20 mM Tris.HCl (pH 7.5), 1 mM EDTA, 150 mM NaCl, 1 mM DTT, 1 mM $NaVO_4$, 100 μM PMSF and protease inhibitor cocktail (Sigma-Aldrich). Equal amounts of total protein were separated by sodium dodecyl sulfate-polyacrylamide gel electrophoresis (SDS-PAGE) on 15% polyacrylamide gels and transferred to polyvinylidene fluoride membranes. Immunodetection was performed with antibodies against phospho-S6 (Ser240/44) (Cell Signaling Technology), LC3 (Nanotool) or p62 (Abnova). For immunofluorescence studies, MEFs were seeded on pre-coated poly-L-lysine coverslips the day before treatment. After treatment, cells were fixed in ice-cold methanol for 15 min followed by blocking in 5% BSA in PBS. Immunostaining for WIPI-II was performed with an antibody kindly provided by Sharon Tooze (London Research Institute-Cancer Research UK) as described elsewhere (3). Confocal images were acquired with an optical slice of 0.8 μm using a 63x oil immersion objective. Secondary antibodies used for this assay were anti-mouse IgG FITC and anti-rabbit IgG Cy5.

T cell surface marker expression. Blood collected from terminally-anaesthetized mice was analysed by flow cytometry. Naive and memory T cell subsets were defined by expression of the following cell surface markers: naive CD4/CD8 T cells = CD44^{Low}CD45RB^{High}CD62L^{High}; memory CD4/CD8 T cells = CD44^{High}CD45RB^{Low}CD62L^{Low}.

Supplemental References

1. Mizushima N *et al.* (2004) In vivo analysis of autophagy in response to nutrient starvation using transgenic mice expressing a fluorescent autophagosome marker. *Mol Biol Cell.* 15: 1101–1111.
2. Foukas LC *et al.* (2006) Critical role for the p110alpha phosphoinositide-3-OH kinase in growth and metabolic regulation. *Nature* 441: 366-370.
3. Polson HEJ *at al.* (2010) Mammalian Atg18 (WIPI2) localizes to omegasome-anchored phagophores and positively regulates LC3 lipidation. *Autophagy* 6: 506-522.

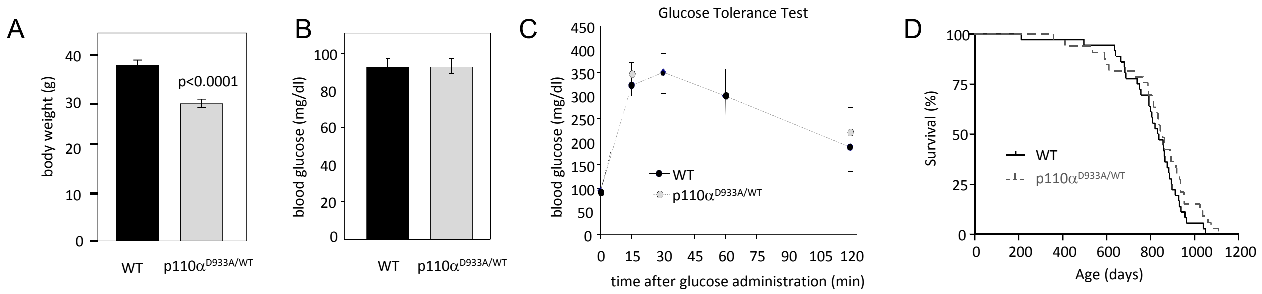

Figure S1. Glucose homeostasis (at middle age) and life span in p110α$^{D933A/WT}$ female mice. (A) Body weight (B) fasting plasma glucose levels (C) glucose tolerance in approximately 500 day old female p110α$^{D933A/WT}$ and WT littermate mice (n=17 per genotype). (D) Survival curves for p110α$^{WT/D933A}$ female mice. Median life span was 858 days in p110α$^{D933A/WT}$ (n=33) *vs* 840 days in WT (n=36) mice. Bars represent mean ± standard error. Statistical comparisons were performed with Student's t test.

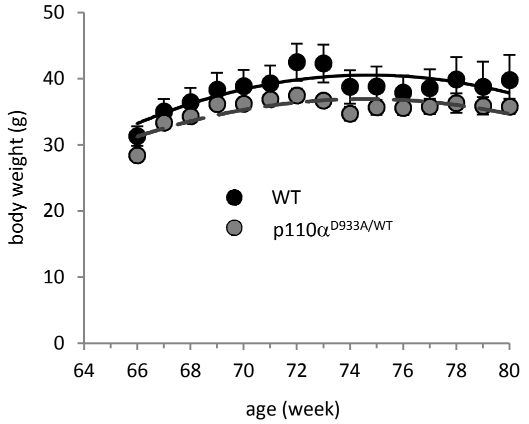

Figure S2. Weight gain in mice fed a high fat diet. Mice were maintained on a high fat diet (45% calories derived from fat) starting at 66 weeks of age and for 14 weeks. Values represent mean ± standard error (n=8 per genotype).

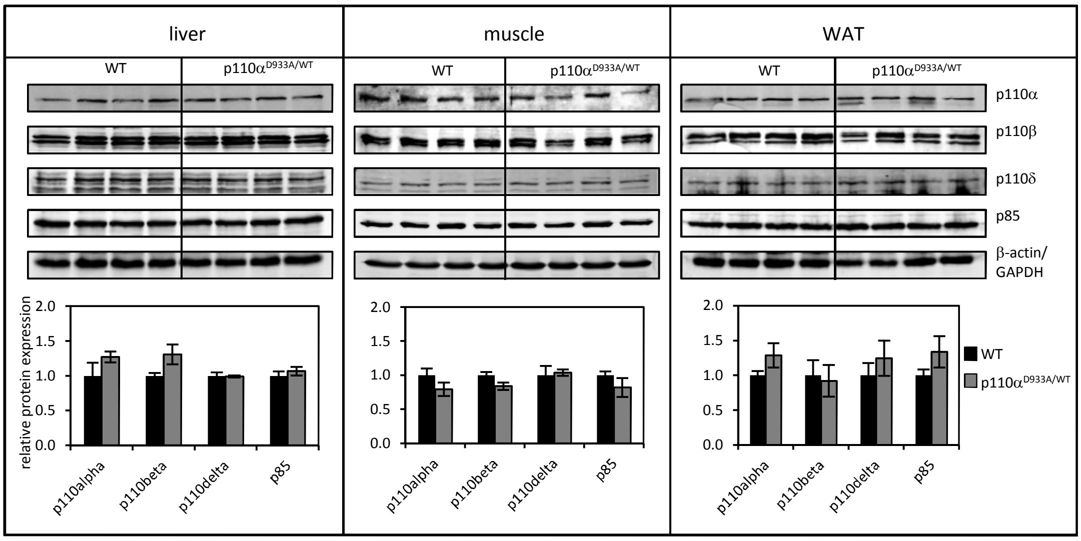

Figure S3. Class IA PI3K isoform expression in tissues from aged p110α$^{D933A/WT}$ mice. The indicated insulin-sensitive tissues were isolated from approx. 80 week old male mice. Tissue homogenates were subjected to quantitative immunoblot analysis using the indicated PI3K isoform-specific antibodies. β-actin and GAPDH were used as loading controls for liver, white adipose tissue (WAT) and muscle (*gastrocnemius*), respectively. Intensities of protein signals were averaged and plotted in bar graphs (mean ± standard error) shown below the respective immunoblots. Each lane was loaded with tissue homogenate derived from an individual mouse (n=4 per genotype).

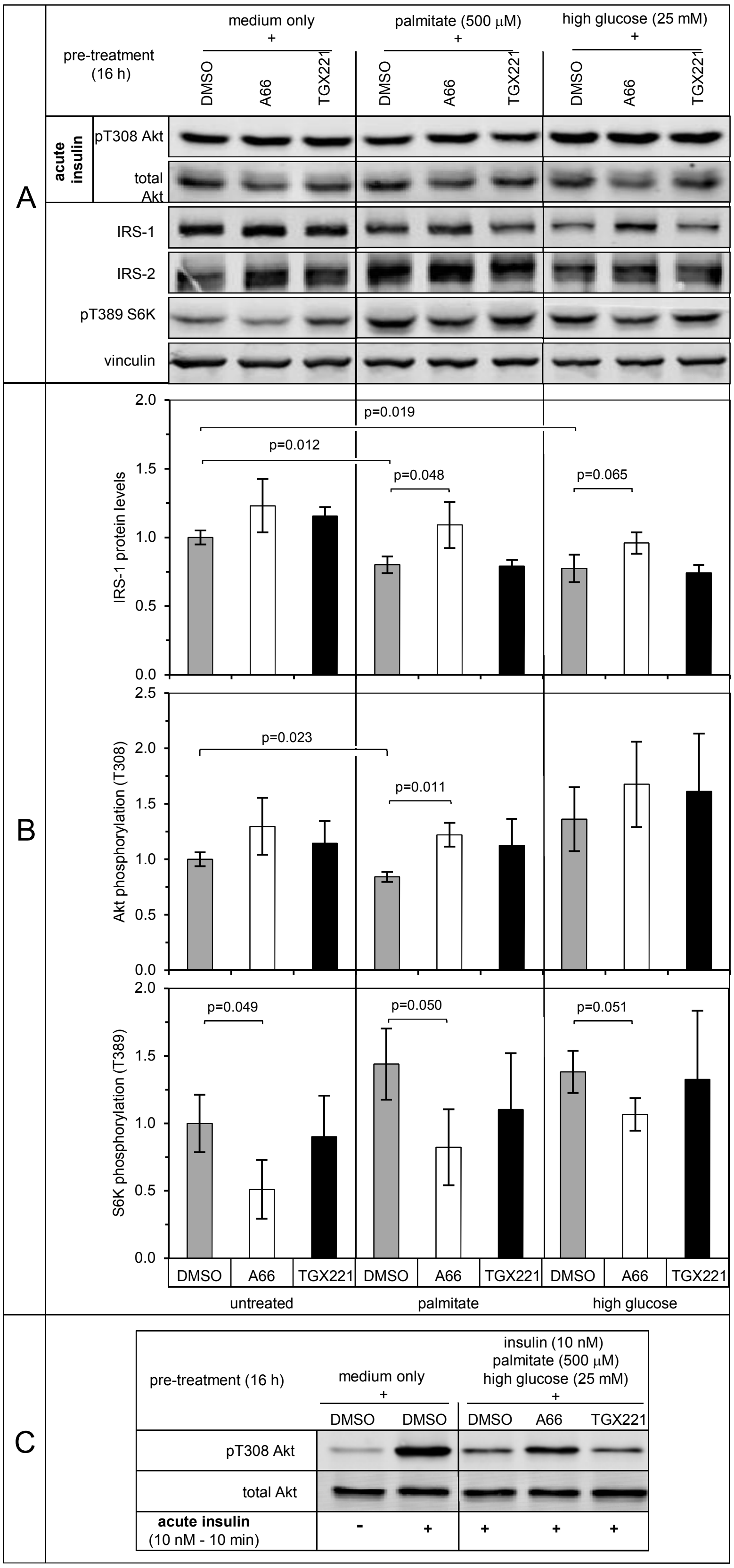

Figure S4. Effect of chronic p110α inhibition on free fatty acid- and high glucose-induced IRS downregulation in C2C12 myotubes. (A) C2C12 myotubes (cultured in

medium with 5.5 mM glucose) were treated with palmitate (500 μM, nominal concentration) or glucose (25 mM) in the presence or absence of A66 (2 μM) or TGX-221 (1 μM) for 16 h. Monolayers were then rinsed with phosphate-buffered saline and lysed either directly for detection of IRS and S6K pT389 phosphorylation or after 1 h culture in serum-free medium and re-stimulation with insulin (10 nM for 10 min) for detection of Akt T308 phosphorylation. Lysates were processed for immunoblot analysis with the indicated antibodies. Panels have been rearranged for clarity, but they are from the same immunoblots. Signal intensity was quantified and normalized to that of vinculin, used as a loading control. (B) shows data (mean ± standard deviation) from three independent experiments. Statistical comparisons were performed with Student's t-test. (C) T308 Akt phosphorylation in cells pre-treated with a combination of insulin, palmitate and high glucose and acutely re-stimulated with insulin, as above.

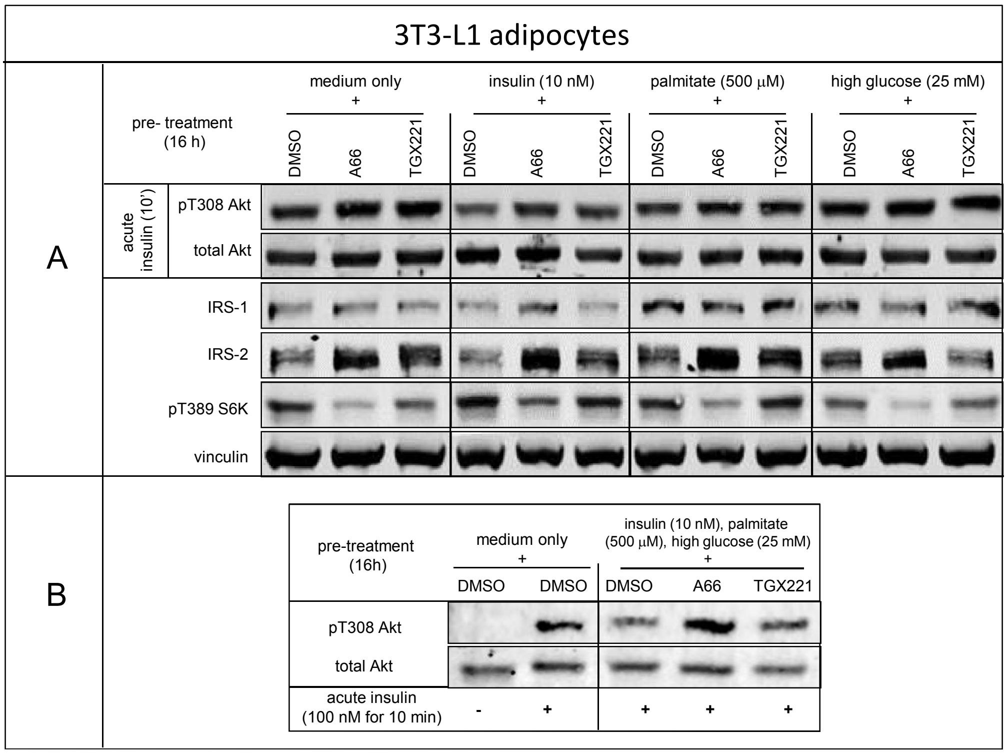

Figure S5. Effect of chronic p110α inhibition on IRS-dependent signaling in 3T3-L1 adipocytes. (A) 3T3-L1 adipocytes (cultured in medium with 5.5 mM glucose) were treated with insulin (10 nM), palmitate (500 μM, nominal concentration) or glucose (25 mM) in the presence or absence of A66 (2 μM) or TGX-221 (1 μM) for 16 h. Monolayers were then rinsed with phosphate-buffered saline and lysed either directly for detection of IRS and S6K pT389 phosphorylation or after 1 h culture in serum-free medium and re-stimulation with insulin (100 nM for 10 min) for detection of Akt T308 phosphorylation. Lysates were processed for immunoblot analysis with the indicated antibodies. Panels have been rearranged for clarity, but they are from the same immunoblots. (B) Akt T308 phosphorylation in cells treated with a combination of insulin, palmitate and high glucose and stimulated with insulin, as above.

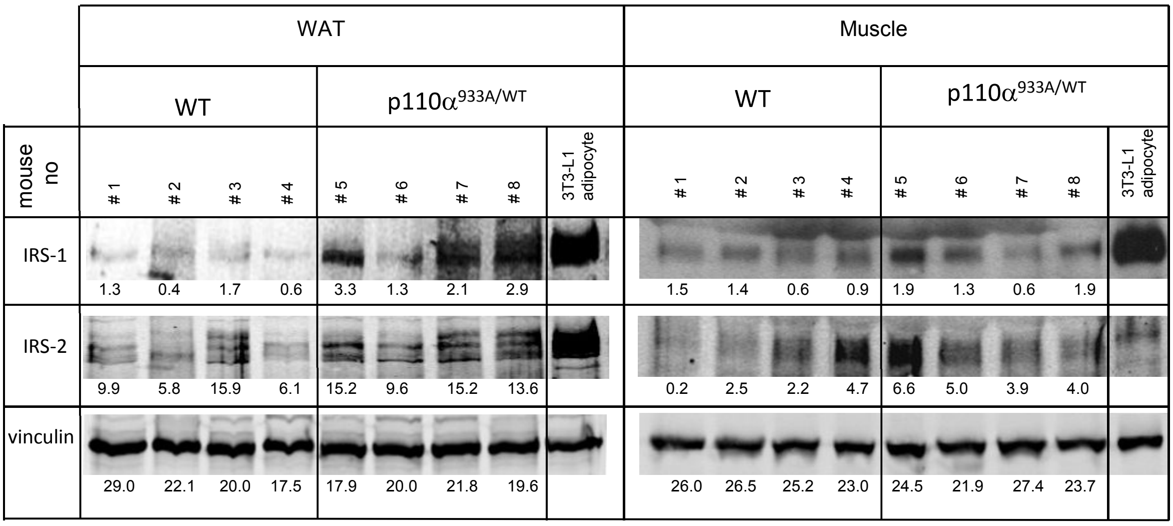

Figure S6. IRS-1 and -2 protein levels in white adipose tissue (WAT) and skeletal muscle of aged mice. Homogenates of epididymal fat pads and *gastrocnemius* muscles isolated from 80 week old p110α$^{D933A/WT}$ male mice and WT littermates (n=4 per genotype) were subjected to immunoblot analysis using the indicated antibodies. Signal intensities are presented for each sample under the respective blots. Graphs of pooled data are presented in Fig 3C.

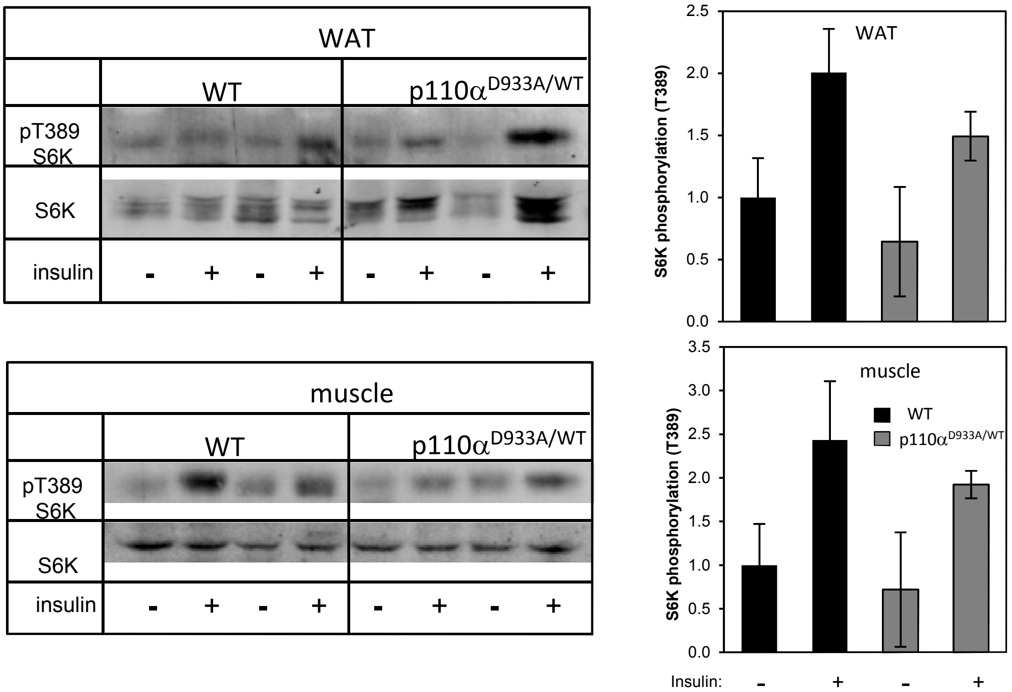

Figure S7. Insulin stimulated S6K Thr389 phosphorylation in white adipose tissue (WAT) and skeletal muscle of aged mice. Homogenates of epididymal fat pads and *gastrocnemius* muscles isolated from 80 week old p110α$^{D933A/WT}$ male mice and WT littermates following injection of 0.25 mU/g insulin through the *inferior vena cava* were subjected to immunoblot analysis using the indicated antibodies. Signal densities were quantified. Values of phosphorylated S6K (Thr389) normalised to total S6K and vinculin, as loading control, are presented in graphs. Bars represent the mean ± standard deviation of S6K phosphorylation in tissues from two mice (n=2) per genotype and treatment shown in the adjacent immunoblots.

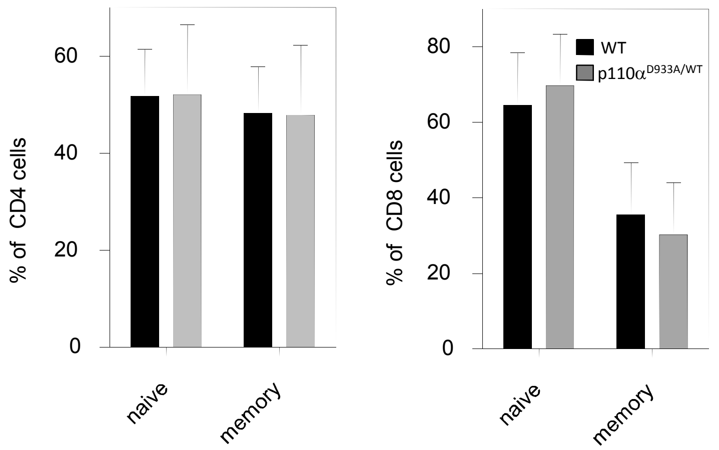

Figure S8. Distribution of T cells in naive and memory cell subsets between WT and p110α$^{D933A/WT}$ mice. Blood derived from 70 week old male mice (n=3 per genotype) was stained with fluorophore-conjugated antibodies and analysed by FACS, as described in Materials and Methods. Bars represent mean ± standard error.

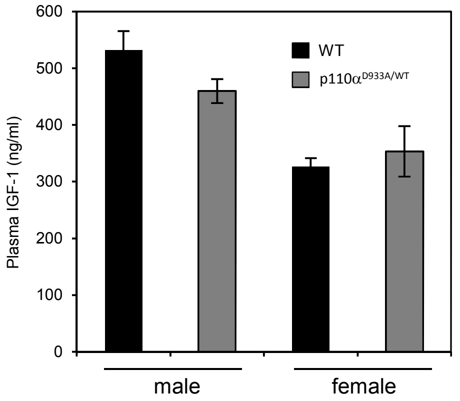

Figure S9. Fasting plasma IGF-1 in young p110α$^{D933A/WT}$ and WT littermate mice. Plasma was isolated from overnight starved 8-9 week old mice. IGF-1 was measured by ELISA (Octeia, IDS). Bars represent mean ± standard error (n=9-11 mice per genotype and sex).

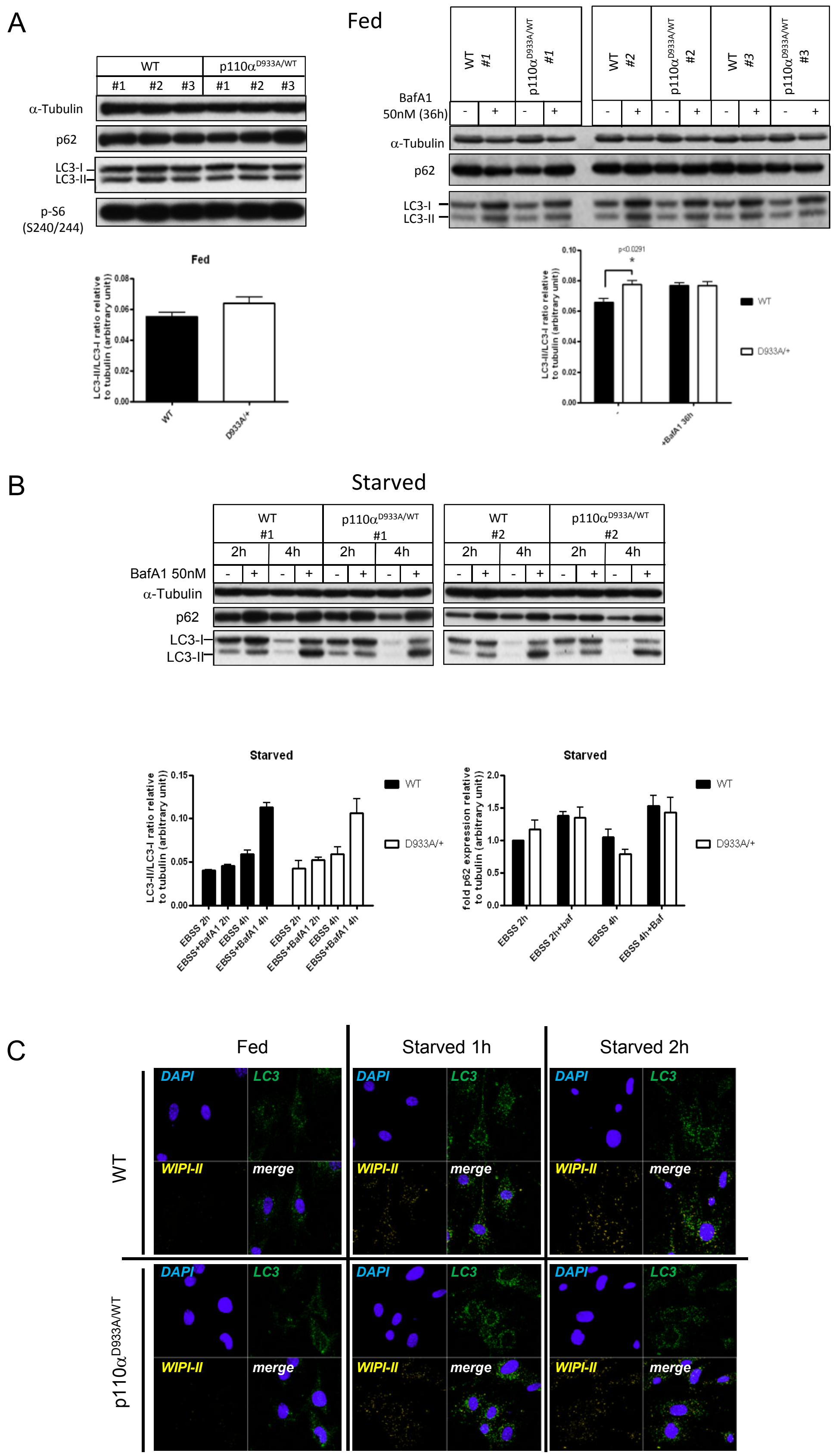

Figure S10. Assessment of autophagy in MEFs derived from p110α$^{D933A/WT}$ embryos. Autophagic activity was assessed using established markers i.e. conversion of LC3-I to LC3-II,

levels of p62 and autophagosome density by WIPI-II staining. (A) WT or p110α$^{D933A/WT}$ MEFs expressing GFP-LC3 transgene were grown in normal condition (Fed). Each lane represents individual embryo-derived MEFs for each genotype (designated as #1-3). LC3-II and LC3-I forms were detected using an LC3 Ab and the levels of p62 expression were monitored. Quantification of the LC3-II/LC3-I ratio and p62 expression relative to the loading control (α-tubulin) is shown in the graph. (B) Same as in (A) but in starved condition (EBSS medium). Each culture was treated for the indicated time with EBSS medium with or without 50 nM Bafilomycin A1 (BafA1). Quantification of the LC3-II/LC3-I ratio and p62 expression relative to the loading control (α-tubulin) is shown in the graph. Bars represent mean ± standard error. Statistical comparisons were performed with Student's t test (C) LC3 and WIPI-II staining in fed *vs* starved condition. LC3 and WIPI-II punctate staining shown in the starved condition is characteristic of autophagy induction and autophagic vacuoles.

Table S1 Survival characteristics of p110α$^{D933A/WT}$ mice. Lifespan is reported (±SEM). N=sample size. Oldest 10% was calculated as mean lifespan of the longest-living 10% of either male or female mice within a genotype.

**Genotype**	**Median**	**Mean**	**Oldest 10%**	**Range**	**N**
p110α$^{D933A/WT}$ female	854	829±31	1080±2	358-1108	33
WT female	835	807±26	1005±3	212-1050	36
p110α$^{D933A/WT}$ male	818	810±53	1153±2	76-1174	26
WT male	799	797±27	1036±1	187-1045	42
p110α$^{D933A/WT}$ male (over 500 days)	852	895±39	1163±11	564-1174	22
WT male (over 500 days)	805	812±23	1037±5	522-1045	41

Table S2 Log-rank test comparisons and hazard ratios of p110α$^{D933A/WT}$ mice. Log-rank test comparisons, x^2 values, statistical significance, hazard ratio and 95% confidence intervals (CI) of the hazard ratio in male and female mice used in the longevity analysis. Statistically significant differences are indicated by bold type.

**Genotype**	**Comparison**	**X^2**	**P**	**Hazard ratio**	**95% CI of ratio**
p110α$^{D933A/WT}$ female	p110α$^{D933A/WT}$ *vs* WT	1.753	0.1855	1.392	0.85-2.27
**p110α$^{D933A/WT}$ female (oldest 10%)**	**p110α$^{D933A/WT}$ *vs* WT (oldest 10%)**	**5.629**	**0.0177**	**11.83**	**1.536-91.02**
p110α$^{D933A/WT}$ male	p110α$^{D933A/WT}$ *vs* WT	3.697	0.0545	1.662	0.99-2.79
**p110α$^{D933A/WT}$ male (over 500 days)**	**p110α$^{D933A/WT}$ *vs* WT**	**6.739**	**0.0094**	**2.046**	**1.19-3.51**
**p110α$^{D933A/WT}$ male (oldest 10%)**	**p110α$^{D933A/WT}$ *vs* WT (oldest 10%)**	**5.3**	**0.0213**	**12.06**	**1.448-100.5**
